# Novel pathogenic characteristics of highly pathogenic avian influenza virus H7N9: viraemia and extrapulmonary infection

**DOI:** 10.1080/22221751.2020.1754135

**Published:** 2020-05-18

**Authors:** Xiao-Xin Wu, Ling-Zhai Zhao, Song-Jia Tang, Tian-Hao Weng, Wei-Gen Wu, Shu-Hao Yao, Hai-Bo Wu, Lin-Fang Cheng, Jian Wang, Feng-Yu Hu, Nan-Ping Wu, Hang-Ping Yao, Fu-Chun Zhang, Lan-Juan Li

**Affiliations:** a State Key Laboratory for Diagnosis and Treatment of Infectious Diseases, National Clinical Research Centre for Infectious Diseases, Collaborative Innovation Center for Diagnosis and Treatment of Infectious Diseases, The First Affiliated Hospital, Zhejiang University School of Medicine, Hangzhou, People’s Republic of China; b Institute of Infectious Diseases, Guangzhou Eighth People’s Hospital, Guangzhou Medical University, Guangzhou, People’s Republic of China; c Plastic and Aesthetic Surgery Department, Affiliated Hangzhou First People’s Hospital, Zhejiang University School of Medicine, Hangzhou, People’s Republic of China; d Department of Stormotologry, Wenzhou Medical University Renji College, Wenzhou, People’s Republic of China

**Keywords:** Highly pathogenic H7N9, viraemia, pathogenic characteristics, exosomes, extrapulmonary infection

## Abstract

The H7N9 virus mutated in 2017, resulting in new cases of highly pathogenic avian influenza (HPAI) H7N9 virus infection. H7N9 was found in a viraemic patient in Guangdong province, China. The present study aimed to clarify the pathogenic characteristics of HPAI H7N9. Virus was isolated from the plasma and sputum of the patient with HPAI H7N9. Liquid phase chip technology was used to detect the plasma cytokines from the infected patient and healthy controls. Mice were infected with strains A/Guangdong/GZ8H002/2017(H7N9) and A/Zhejiang/DTID-ZJU01/2013(H7N9) to observe the virus’s pathogenic characteristics. Serum and brain tissue were collected at 2, 4, and 6 days after infection. The viruses in serum and brain tissue were detected and isolated. The two strains were infected into A549 cells, exosomes were extracted, and virus genes in the exosomes were assessed. Live virus was isolated from the patient’s plasma. An acute cytokine storm was detected during the whole course of the disease. In animal experiments, A/Guangdong/GZ8H002/2017(H7N9) was more pathogenic than A/Zhejiang /DTID-ZJU01/2013(H7N9) and resulted in the death of mice. Live virus was isolated from infected mouse serum. Virus infection was also detected in the brain of mice. Under viral stress, A549 cells secreted exosomes containing the entire viral genome. The viraemic patient was confirmed to have an HPAI H7N9 infection. A/Guangdong/GZ8H002/2017(H7N9) showed significantly enhanced toxicity. Patient deaths might result from cytokine storms and brain infections. Extrapulmonary tissue infection might occur via the exosome pathway. The determined pathogenic characteristics of HPAI H7N9 will contribute to its future treatment.

## Introduction

In February 2013, the avian influenza A H7N9 virus was identified as causing human infection, with high mortality and morbidity. To date, five waves of H7N9 infection have caused more than 1500 severe human infections and the mortality remains high, with more than 600 deaths [1]. The threat of a fifth wave of H7N9 virus to public health was more serious than that of the first four waves. The potential mechanism of this phenomenon may be that the later H7N9 viruses had a stronger ability to bind human receptors than the early isolates [2]. Before 2017, the H7N9 viruses comprised low-pathogenic avian influenza A (LPAI) H7N9 viruses. The H7N9 virus was spread in poultry, waterfowl, and migratory birds. Patients with H7N9 infection commonly had a history of exposure to sick and dying poultry. No evidence of human-to-human transmission was found. In the beginning of 2017, a novel highly pathogenic avian influenza A virus (HPAI) H7N9 was identified in human cases [3]. Since the first case of HPAI H7N9 infection, these HPAI H7N9 strains have caused human infection in 8 provinces [4] in China, and 32 human cases were found to be caused by the HPAI H7N9 virus. Meanwhile, the HPAI H7N9 virus was continuously detected in poultry or environmental samples in 14 provinces. Approximately 60,000 poultry have died from HPAI H7N9 virus infections. The rapid growth of human cases and the geographical expansion of the HPAI H7N9 virus pose a great challenge to public health.

In a previous study of H5N1 virus infection, viraemia was detected in 82% of the fatal cases, but absent in nonfatal cases. High viral load, and the resulting intense inflammatory responses, are central to influenza H5N1 pathogenesis. Individuals with detectable H5N1 RNA in their blood also had higher pharyngeal viral loads than those without evidence of H5N1 RNA, suggesting that the presence of viral RNA in blood reflects an overall high viral burden, which commonly leads to high mortality [5]. Virus RNA in serum has also been detected in patients with severe pandemic H1N1 2009 infection [6]. In our previous study, H7N9 was only detected in the respiratory tract and faeces. Reverse transcription polymerase chain reaction (RT-PCR) for H7N9 was negative in the cerebrospinal fluid, urine, or blood of all patients [7]. However, in another centre in Shanghai, virus RNA was detected from plasma in many patients, but with no correlation with clinical outcome. Viral RNA might represent non-infectious virus rather than infectious virus particles. No live viruses were isolated in the blood of these patients [8].

Genetic changes to viruses might change their behaviour and pathogenic characteristics [9]. We treated a 63-year-old male patient with HPAI H7N9 infection. This patient had onset symptoms of high fever (42°C), sore throat, cough, palpitations and shortness of breath on day 9. The patient went to the Guangzhou Eighth People’s hospital and was admitted. This patient was severely infected and died 3 days after admission. We isolated H7N9 virus from the patient’s plasma. Isolation of virus was important to prove whether the virus disseminates beyond the respiratory tract. HPAI H7N9 caused severe viraemia, which might result in intense inflammatory responses, contributing to the high mortality. When live virus was isolated in the blood, virus dissemination beyond the respiratory tract became possible.

Exosomes play an important role in viral infection [10]. Studies have shown that exosomes often carry viral RNA and proteins [11]. Moreover, studies have shown that the exosomes can carry intact HIV, HAV, HCV, and Zika viruses, causing their transmission and infection [12–16]. Thus, exosomes have been confirmed as a route of virus transmission [17]. The H7N9 virus might also spread through via exosomes. Therefore, the present study aimed to determine the novel pathogenic characteristics of highly pathogenic H7N9.

## Materials and methods

### Ethical approval

The work was carried out in accordance with ethical guidelines of the Guangzhou Eighth People’s hospital (Ethical approval No. 20170178). Sputum and plasma specimens were collected daily after admission. The patient’s plasma remaining after routine testing was stored in a bio-sample banker. The normal plasma were collected from 10 volunteers (half male and half female). The median age of these volunteers was 26 (interquartile range 23–28 year) year old. These volunteers came to the First Affiliated Hospital of Zhejiang University School of Medicine for regular physical examination. After the physical examination, these volunteers were confirmed healthy. The remaining plasma after routine testing was stored and used in our study.

### Animal experimental ethical statement

Female specific-pathogen-free (SPF) BALB/c mice, aged 6–8 weeks old, were purchased from the Shanghai Laboratory Animal Center, China. All animal studies were performed in accordance with the Guide for the Care and Use of Laboratory Animals of Zhejiang Province and the study was approved by the Ethics Committee of the First Affiliated Hospital, College of Medicine, Zhejiang University (Ethical approval No. 2017-402-1). All experiments with H7N9 virus were performed in a biosafety level 3 laboratory approved by the First Affiliated Hospital, School of Medicine, Zhejiang University (Permit No. 2015-15).

### Cells and viruses

The Madin-Darby canine kidney cell line (MDCK) and A549 cell line (adenocarcinomic human alveolar basal epithelial cells) were obtained from the ATCC (Rockville, MD, USA). The cell lines were propagated in growth medium containing Dulbecco’s modified Eagle’s medium (DMEM; Cat#11965092, Gibco, Grand Island, NY, USA) supplemented with 10% foetal bovine serum (FBS; Cat#10100147, Thermo Fisher Scientific, Waltham, MA, USA) at 37°C and 5% CO2. The A/Guangdong/GZ8H002/2017(H7N9) virus used in this study was isolated from a patient in Guangzhou, China, in 2017 (GenBank: MF455313- 455320). We isolated this virus from the patient’s plasma. The A/Zhejiang /DTID-ZJU01/2013(H7N9) virus was isolated from a patient in Zhejiang Province, China, in 2013 [18]. Virus stocks were propagated in the allantoic cavities of 9-day-old SPF embryonated chicken eggs at 37°C for 72 h. The allantoic fluid was harvested and tested using a haemagglutinin HA assay. Aliquots of the allantoic fluid containing the virus were stored at −80°C until further use.

### Deep sequencing of the H7N9 virus isolated from the plasma

The viral RNA of the H7N9 virus was extracted using Trizol (Cat#15596-026, Invitrogen, Waltham, MA, USA) method. The concentration, purity, and integrity of total viral RNA were detected using a NanoDrop 2000 instrument (Thermo Fisher Scientific, Waltham, MA, USA) and an Agilent 2100 Bioanalyzer (Agilent Technologies, Santa Clara, CA, USA). The sequencing library was constructed using TruSeq Stranded Total RNA with Ribo-zero Gold (Cat# RS-122-2301, Illumina, San Diego, CA, USA), and the process was conducted according to the manufacturer’s protocol. The library was constructed as follows: after digesting ribosomal RNA with Ribo-zero, the RNA was broken into fragments of about 350 nt using Fragment High Mix, and the sequencing library was constructed by synthesizing and purifying cDNA, terminal repair, adding poly-A tail, ligating sequencing connectors, and PCR amplification. After quality checking, the library was sequenced using an Illumina Hiseq Xten sequencer. The sequencing strategy generated 150 bp paired ends (PE150).

Clean reads were obtained through data quality control and filtering of raw sequencing data using the NGSQC toolkit [19]. Clean Reads were compared to the reference genome (A/Zhejiang/DTID-ZJU01/2013(H7N9) virus) using BWA software [20]. Based on the results of the comparison between the samples and the reference genome, single nucleotide polymorphisms (SNPs) and insertion/deletion (INDEL) sites were detected using the Freebayes, and the snpEff software [21] was used for functional annotation of SNPs and INDELs. The screening criteria of SNPs and INDELs were that the number of covered reads was greater than five and the quality value was greater than 20.

### The viral replication capability

The TCID50 (Fifty percent tissue culture infective dose) assay was performed as described previously[22]. The virus replication capability were valued using embryonated chicken eggs and A549 cells models. The two strains of viruses (both with the amount of 100 TCID50) were injected into 9-day-old SPF embryonated chicken eggs and cultured in incubator at 37°C for 72 h. The replication capability of the two strains of viruses were measured by the quantity of RNA and TCID50 titre.

A549 cells were cultured in DMEM supplemented with 10% FBS at 37°C and 5% CO_2_. When the cell density reached 70­–80%, the cells were washed three times with PBS to remove the bovine serum. The cells were then infected with the viruses (MOI=0.1). After the adsorption for 2 h, the cells were washed three times with PBS. DMEM supplemented with 1% foetal bovine albumin and 1 µg/ml L-l-tosylamide-2-phenylethyl chloromethyl ketone (TPCK) was added to the culture A549 cells for 48 h when culturing the ZJU01 strain. DMEM supplemented with 1% foetal bovine albumin was added to the culture A549 cells for 48 h while culturing the GZ8H002 strain. The virus culture supernatant was measured by the quantity of RNA and TCID50 titre.

After culture for 48 h, the cells were washed twice with PBS slightly. Cells were then fixed with 4% paraformaldehyde for 20 min. After washed twice with PBS slightly, cells were penetrated with 0.1% Triton X-100 for 20 min. Cells were again washed, blocked for 30 min with 3% bovine serum albumin (BSA; Cat#H1130, Solarbio, Tongzhou, Beijing, China) and incubated sequentially with 1:400 dilution of mouse anti-influenza A virus nucleoprotein antibody (Cat#ab128193, Abcam, Cambridge, MA, USA) for 1 h. After washing 5 times with PBS, the Alexa Fluor 488 conjugated goat anti-mouse IgG (Cat#ab150133, Abcam, Cambridge, MA, USA) with 1:500 dilution was used as the secondary antibody. After washing 5 times with PBS, DAPI (Cat#C0060, Solarbio, Tongzhou, Beijing, China) was used as the nuclear counterstain. After washing 5 times with PBS, the cells were observed under the fluorescence microscope.

### Virus inoculation

Mice were inoculated intranasally with 50 µl 10^4^ TCID50 A/Guangdong/GZ8H002/2017(H7N9) or the A/Zhejiang/ DTID-ZJU01/2013(H7N9) virus. Phosphate-buffered saline (PBS; Cat#20012500BT, Gibco, Grand Island, NY, USA) of the same volume was given to the mice in the control group. Mice were observed for illness, weight loss, and death post-infection. Then, the mice were sacrificed, and their serum was collected at 2, 4, and 6 days post-infection (dpi) . The lung and brain were removed. Part of the organ was fixed in 10% buffered formalin, while the other part was used to detect virus levels using quantitative PCR. An H7N9 nucleic acid quantitative detection kit (Cat#Z-RR-0309-02) and Influenza virus A Real time RT-PCR kit (Cat#RR-0051-01) were purchased from Zhijiang biological technology Co., Ltd. Shanghai, China.

### Isolation of the virus from mouse serum

The sera collected at 2, 4, and 6 dpi were used to isolate the virus. About 100 µl of serum was injected into the allantoic cavities of 9-day-old SPF embryonated chicken eggs. The embryonated chicken eggs were cultured in an incubator at 37°C for 72 h. The allantoic fluid was harvested and tested using an HA assay. Then 100ul serum from the 6 dpi infected mice was used to detect virus levels using quantitative PCR. Meantime, we collected 1.2 ml of serum from the 6 dpi infected mice and diluted in 40 ml of PBS. The dilution was subjected to ultra-centrifugation at 120000 × *g* for 3 h in a Beckman Coulter Optima XPN-80 Ultracentrifuge (Beckman Coulter, Brea, CA, USA). The pellet of virus was resuspended by 200 µl of PBS. The viruses were observed using a Tecnai Spirit 120 kv transmission electronic microscope to analyse their structure.

### Virus titres and the histopathology of organ tissue

The amount of virus was evaluated using quantitative PCR. Organ tissue was prepared for haematoxylin eosin (HE) staining. Immunohistochemical (IHC) assays were also conducted. Paraffin sections of organs were de-waxed and then subjected to heat treatment in citrate buffer, and endogenous peroxidase activity was quenched using 0.3% H_2_O_2_ in methanol. Sections were blocked for 1 h with 3% bovine serum albumin (BSA; Cat#H1130, Solarbio, Tongzhou, Beijing, China) in PBS and incubated sequentially overnight at 4°C with 1:200 dilution of polyclonal rabbit anti-H7N9 antibodies(Cat#GTX125989, GeneTex, Irvine, CA, USA). Antibody binding was detected using EnVision System reagents (Cat#K5007, DAKO, Glostrup, Denmark). All slides were counterstained with haematoxylin.

### Cytokines and chemokines detection

The plasma of the patient was collected from the patient with HPAI H7N9 infection. Ten normal plasma samples were collected from the healthy volunteers. The cytokines and chemokines in human plasma was measured using the Bio-Plex Pro™ Human Cytokine Screening Panel, 48-Plex (Cat#12007283, Bio-Rad, Hercules, CA, USA). Serum samples from the mice were detected using Bio-Plex Pro™ Mouse Cytokine Grp I Panel 23-Plex (Cat#M60009RDPD, Bio-Rad, Hercules, CA, USA). The detection was based on the Bio-Plex 200 system (Bio-Rad) and performed according to the manufacturer instructions.

### Exosome isolation and detection

The culture of the two strains of viruses in A549 cell model was described in the previous part. After 48 h of culture, the culture supernatant was collected by centrifugation at 500 ×* g* to remove the cells. Then 40 ml virus culture supernatant was obtained. The supernatant was then subjected to ultra-centrifugation at 120000 ×* g* for 3 h in Beckman Coulter Optima XPN-80 Ultracentrifuge. The pelleted exosomes were resuspended in 200 µl of PBS. The exosomes were then observed using a Tecnai Spirit 120 kv transmission electronic microscope.

### The purification of exosomes and identification of H7N9 genes

To separate the virus from the exosomes, exosomes were purified using an Exosome-human CD63 Isolation kit (Cat#10606D, Invitrogen, Waltham, MA, USA). The exosomes were bound to the magnetic beads marked by CD63. The viruses were separated by washing with solution buffer. Then, 40 µl of magnetic beads were washed with 200 µl of isolation buffer (PBS with 0.1% BSA). The pre-enriched exosome solution was titrated with isolation buffer for the magnetic beads and mixed well. The beads were incubated overnight for 22 h at 4°C with mixing. The bead-bound exosomes were washed by adding 400 µl of isolation buffer and mixed gently by pipetting. The tube was placed on a magnet for 1 min and the supernatant was discarded. These steps were repeated three times. The purification of exosome was verified, with no H7N9 detected in supernatant (Cq > 38 was considered negative). The last supernatant was collected and the bead-bound exosomes were resuspended by 300 µl of isolation buffer. The identification of H7N9 genes in the exosome was performed using an H7N9 nucleic acid quantitative detection kit. Then, 100 µl of bead-bound exosomes solution was injected to the allantoic cavities of 9-day-old SPF embryonated chicken eggs and cultured at 37°C for 72 h. The allantoic fluid was harvested and tested using an HA assay.

### Statistical analysis

Statistical analyses of data for weight and survival rate were performed using Graphpad Prism 5 (GraphPad Software, Inc. La Jolla, CA, USA). Statistical analyses of the data for cytokines and chemokines were conducted using the Mann–Whitney test. Statistical analyses of the data for H7N9 gene expression in A549, embryonated chicken egg and mouse serum were conducted using a one-way analysis of variance (ANOVA) test with Tukey’s multiple comparison test. Statistical analyse of the data for H7N9 gene expression in exosome-enclosed viral particle was conducted using paired t test. Statistical analyses of the data for H7N9 gene expression in the brain and lung leachates were conducted using a two-way ANOVA test with Bonferroni post-tests. The one-way analysis of variance (ANOVA) test with Tukey’s multiple comparison test was used to compare the TICD50 titre of H7N9 virus isolated from the exosomes. The values are presented as means ± SD for the indicated sample sizes. *P*-values < 0.05 were considered statistically significant.

## Results

### High level of cytokines and chemokines in the patient

**Figure 1.**
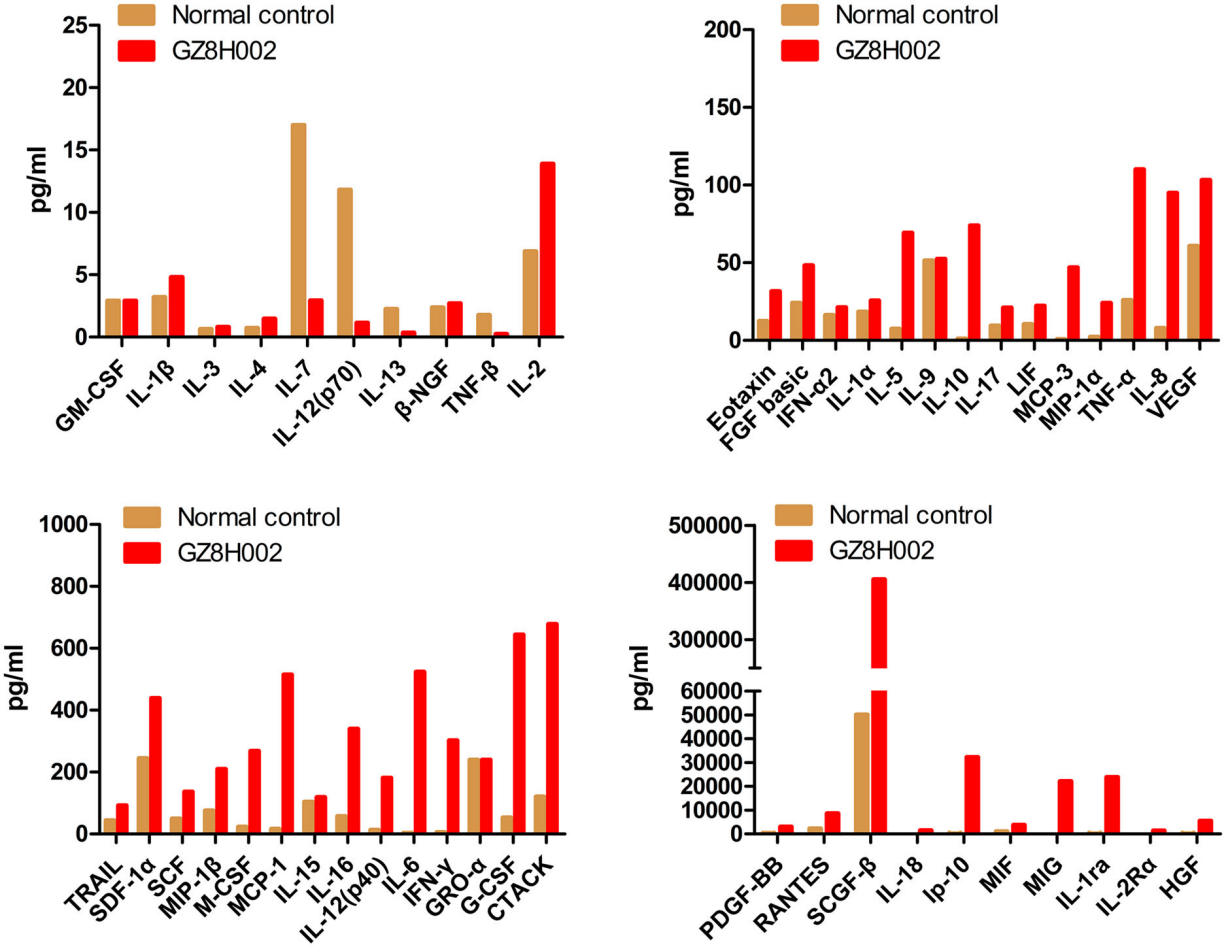
The clinical characteristics of the HPAI H7N9 virus. The cytokines in the plasma of the patient with HPAI H7N9. The levels of ten cytokines and chemokines, including MIG, IL-1ra, IL-6, IP-10, IL-10, IFN-γ, MCP-3, IL-18, HGF, and MCP-1, were higher in the patient (> 30-fold) compared with those in the control group.

The patient suffered a cytokine storm in the hospital. Forty-eight cytokines and chemokines were tested. Among them, the levels of 32 cytokines and chemokines including IL-1ra, IL-6, IP-10, IL-10, IFN-γ, MCP-3, IL-18, HGF, MCP-1, MIG, IL-2Rα, IL-12(p40), IL-8, MIP-1α, PDGF-BB, G-CSF, CTACK, MIF, SCF, HGF, IL-16, SCGF-β, RANTES, TNF-α, FGF basic, IL-2, IL-17, MIF, MIP-1β, SCF, SDF-1α, and TRAIL were elevated in the patient. The levels of ten cytokines and chemokines including MIG, IL-1ra, IL-6, IP-10, IL-10, IFN-γ, MCP-3, IL-18, HGF, MCP-1 were dramatically higher in this patient (Figure 1) compared with those in the healthy controls.

### Gene mutation and virus replication capability

**Figure 2.**
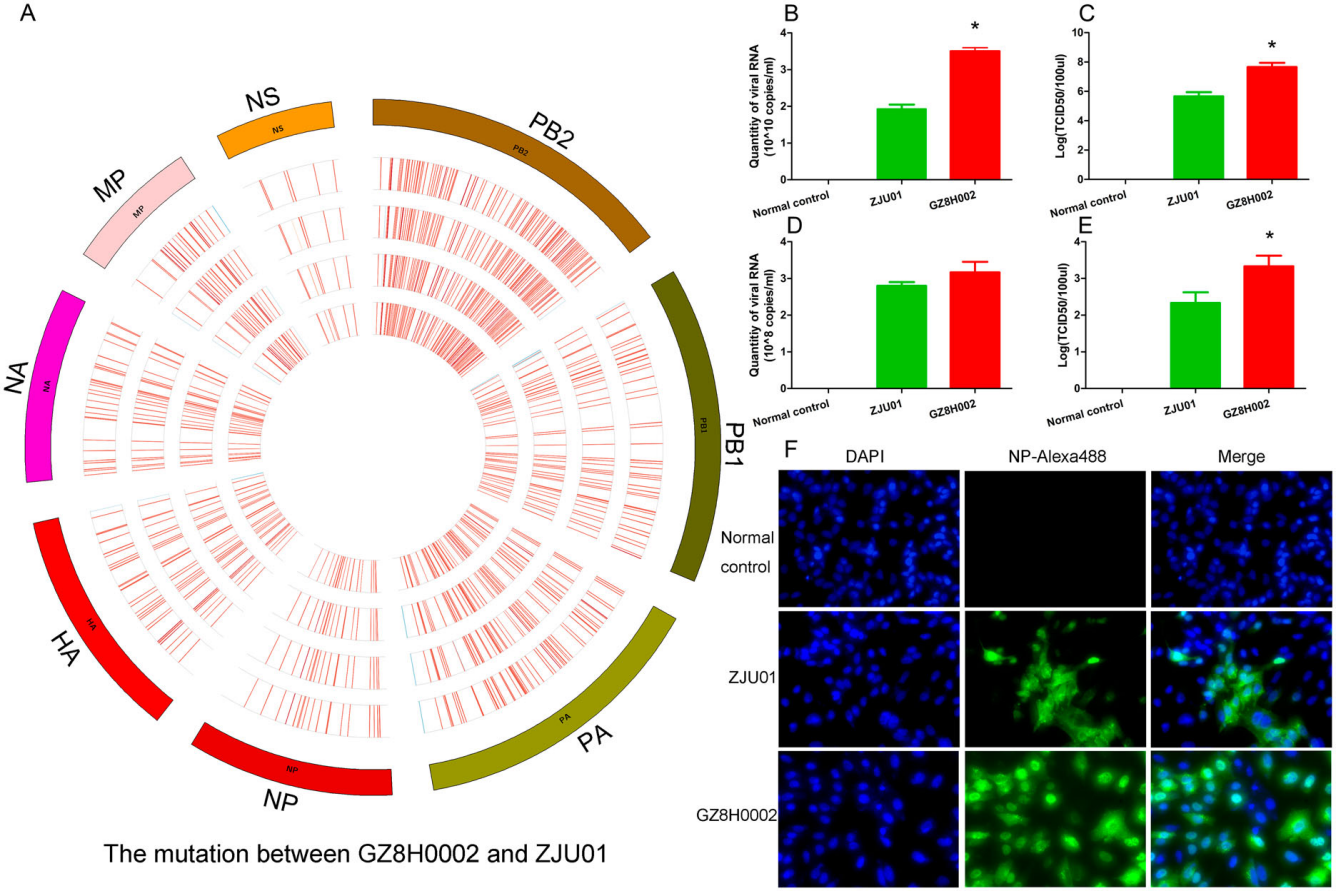
Gene mutation and virus replication capability. A: A circos diagram showing the gene mutations. The genome of A/Zhejiang /DTID-ZJU01/2013(H7N9) was used as a reference genome. The circos diagram was constructed to show the mutations in the different genes (HA, NA, PA, PB1, PB2, NP, MA, and NS). In the circos diagram, the innermost circle represents the virus isolated from the plasma. The other circles represent the viruses isolated from the sputum collected at different times. The marked lines show the mutation sites. B-C: The virus replication capability in embryonated chicken eggs. Replication capability was measured by the quantity of RNA and TCID50 titre. D-E: The virus replication capability in embryonated A549 cells. Replication capability was measured by the quantity of RNA and TCID50 titre. F: Cell fluorescence map. The virus replication capability was indicated by fluorescence intensity.

The HPAI virus could be isolated from both the sputum and the plasma. Deep sequencing of the HPAI virus isolated from the sputum and the plasma were performed. The genome of ZJU01 strain was used as the reference genome. A circos diagram was constructed to present the mutations in the different genes (HA, NA, PA, PB1, PB2, NP, MP, NS). In the circos diagram, the innermost circle represents the virus isolated from the plasma. The other circles represent the virus isolated from the sputum collected at different times. The HPAI virus isolated from the sputum (three sputum samples from the same patient) and the plasma were highly similar. There were only rare mutations between them. However, the genes of the HPAI virus had many mutation in the eight viral genes compared with ZJU01 strain (Figure 2A).

We further compared the replication ability of these two virus. When cultured in embryonated chicken eggs, the replication ability of A/Guangdong/GZ8H002/2017(H7N9) virus was better than that of A/Zhejiang /DTID-ZJU01/2013(H7N9). The quantity of RNA and the TCID50 titre of A/Guangdong/GZ8H002/2017(H7N9) was higher than those of A/Zhejiang /DTID-ZJU01/2013(H7N9) (Figure 2B,C). When cultured in A549 cells, the quantity of RNA of A/Guangdong/GZ8H002/2017(H7N9) virus was similar to that of A/Zhejiang /DTID-ZJU01/2013(H7N9) (Figure 2D). However, the TCID50 titre of A/Guangdong/GZ8H002/2017(H7N9) virus was higher than that of A/Zhejiang /DTID-ZJU01/2013(H7N9) (Figure 2E). Thus, the viral replication capability of A/Guangdong/GZ8H002/2017(H7N9) strain was significantly higher than that of A/Zhejiang /DTID-ZJU01/2013(H7N9) (Figure 2).

### Key amino acid mutations occurring in A/Guangdong/GZ8H002/2017(H7N9)

To determine whether the A/Guangdong/GZ8H002/2017(H7N9) strain had acquired any key molecular substitutions associated with increased virulence and transmissibility in mammals, or with antiviral drug resistance, we performed deep sequencing of the HPAI H7N9 virus. As shown in Table 1, the A/Guangdong/GZ8H002/2017(H7N9) virus had acquired a four-amino-acid insertion in the HA cleavage site. The very important position L217Q in the HA protein leading to antigenicity change was missing in A/Guangdong/GZ8H002/2017(H7N9) [23]. The A/Guangdong/GZ8H002/2017(H7N9) virus has also acquired a significant mutation, R289 K, in the NA protein that leads to drug resistance [24–27]. The K526R and M535L mutations conferring adaptations for mammalian infection have occurred in the PB2 protein of the A/Guangdong/GZ8H002/2017(H7N9) virus. However, almost no D701N mutations were detected in this HPAI virus [4]. In the PA protein, we found two major mutations, V100A and S409N, and in the NP protein we found V33I [4], which cause the virus to be able to infect humans and well as avians. Moreover, the V100A mutation was found to be related to the fatality of H7N9 infection [28,29].

**Table 1.** Key molecular markers of A/Guangdong/GZ8H002/2017(H7N9) virus that differ from the A/Zhejiang/DTID- -ZJU01/2013(H7N9) virus.

Gene	Function	Mutation	Depth	Purity
HA^a^	Cleavage peptides	PEVPKRKRTAR↓G	283,686	99.98%
	Antigenicity change (Immune escape)	L217Q	564,259	99.9%
NA^b^	Reduce drug sensitivity	R289K	646,718	99.96%
PB2	Enhance viral transcription and replication in cells	K526R	532,901	99.96%
	Restore polymerase activity	M535L	492,611	99.95%
	Increase virulence in mammalian models	D701N	744,572	0.05%
PA	Host signature amino acids (avian to human)	V100A	380,980	99.9%
		S409N	457,038	99.97%
NP	Host signature amino acids (avian to human)	V33I	303,797	99.97%

^a^The H7 numbering system was used.

^b^The N9 numbering system was used.

### The pathogenic characteristics of highly pathogenic H7N9 in mice

#### The weight change and survival status of mice

**Figure 3.**
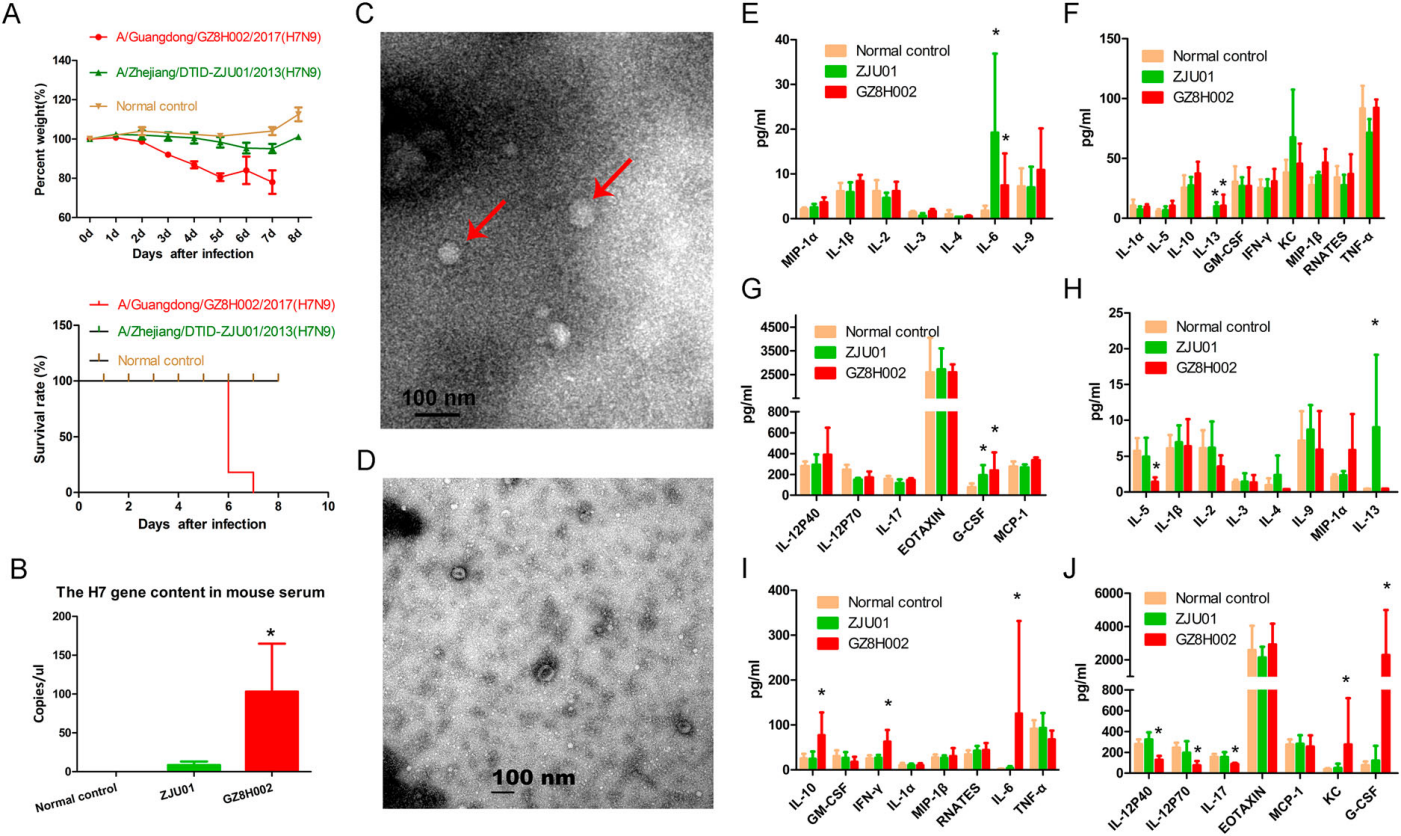
The pathogenic characteristics of HPAI H7N9 in mice. **A:** The weight change and survival curves of mice. The mice challenged with A/Guangdong/GZ8H002/2017(H7N9) all died by 7 days post-infection. The mice challenged with A/Zhejiang/DTID-ZJU01/2013(H7N9) presented with mild illness and weight loss in the first 7 days. In the following period, these mice recovered with an increase in body weight. The mice in normal control group were normal during the entire observation period with no obvious weight loss or illness. B: The H7 gene content in mouse serum. At 6 dpi, the H7 gene in the serum form mice infected with GZ8H002 strain was significantly higher than that from mice infected with ZJU01 strain. C: The typical structure of the virus observed in the serum of mice. The virus was observed using a Tecnai Spirit 120 kv transmission electronic microscope(68,000 × magnification). The typical structure of the virus is indicated by a red arrow. D: The serum of mice observed using the transmission electronic microscope. E-G: The cytokine levels in H7N9-infected mice at 2 dpi. H-J: The cytokines level in H7N9-infected mice at 6 dpi. dpi: days post infection.

We observed illness, weight loss, and death in the mice after H7N9 challenge. Various amounts of weight change were noted in all three groups (Figure 3A). The mice challenged with A/Guangdong/GZ8H002/2017(H7N9) presented a relatively acute clinical process, and showed inactivity, ruffled fur, and poor appetite post-infection. The status of the mice gradually deteriorated over the following days. By day 6 post-infection, the remaining mice presented with severe signs of respiratory disease, including respiratory distress and further lack of appetite, and nearly 20% weight loss. All of the mice were dead by day 7 post-infection. The mice challenged with A/Zhejiang/DTID-ZJU01/2013(H7N9) presented with mild illness and weight loss in the first 7 days. Thereafter, the mice gradually recovered, with an increase in body weight. The mice in normal control group were healthy during the entire observation period with no obvious weight loss or illness.

### Virus isolation from mouse serum

We isolated the H7N9 virus from the mouse serum. In the A/Guangdong/GZ8H002/2017(H7N9) infection mice, the live virus was detected persistently in the serum until death (Table 2). The serum collected at 2 dpi from the mice infected with A/Zhejiang/DTID-ZJU01/2013(H7N9) contained live virus (Table 2). In the following days of infection, live virus was not detected in serum. At 6 dpi, the H7 gene in the serum was detected using quantitative PCR. The H7 gene in the serum from mice infected with GZ8H002 strain was significantly higher than that from mice infected with ZJU01 strain (Figure 3B). At 6 dpi, the typical structure of virus was observed in the serum from mice infected with GZ8H002 strain by transmission electronic microscope (Figure 3C). While using the serum from mice infected with ZJU01 strain, the typical structure of the virus could not be observed by transmission electronic microscopy (Figure 3D).

**Table 2.** The H7N9 virus isolated from serum and tissue leachate of mice.

H7N9 virus strain	Sample	2 dpi	4 dpi	6 dpi
A/Guangdong/GZ8H002/2017(H7N9)	Serum	+	+	+
	Lung	+	+	+
	Brain	−	−	+
A/Zhejiang/DTID-ZJU01/2013(H7N9)	Serum	+	−	−
	Lung	+	+	+
	Brain	−	−	−

Note: +: Positive −: Negative; dpi, days post infection.

### The cytokine changes in H7N9 infected mice

We collected the serum of mice infected with A/Zhejiang/DTID-ZJU01/2013(H7N9) and A/Guangdong/GZ8H002/2017(H7N9) in early (2 dpi) and late stages (6 dpi) of infection and detected the serum cytokine levels. In the early stage of infection, IL-6, G-CSF, and IL-13 levels were high in the A/Zhejiang/DTID-ZJU01/2013(H7N9) and A/Guangdong/GZ8H002/2017(H7N9) infection groups (Figure 3E-G). In the late stage of infection, IL-6 and G-CSF levels in the A/Zhejiang/DTID-ZJU01/2013(H7N9) infection group had decreased to normal levels. The IL-13 level remained higher than that in the normal control. The mice infected with A/Guangdong/GZ8H002/2017(H7N9) continued to suffer from the cytokinemia. The levels of IL-6, IL-10, G-CSF, IFN-γ and KC were significantly higher than those in the normal control group (Figure 3 H-J).

### Histopathology of lung tissue and the lung viral titre

**Figure 4.**
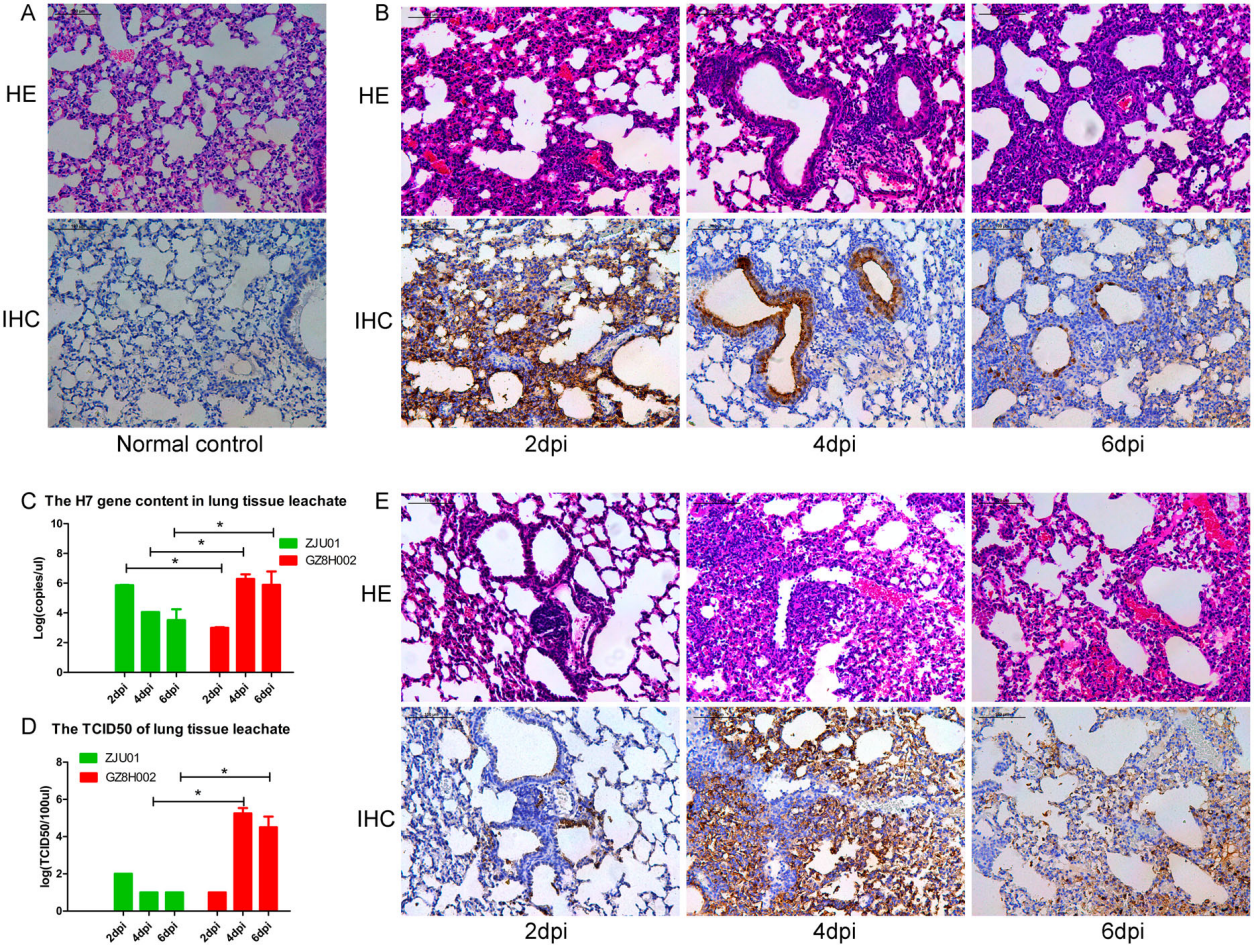
Histopathology of lung tissue and the lung viral titre. A: HE staining and immunohistochemical staining of the lungs of normal mice. B: The lungs of mice challenged with A/Zhejiang/DTID-ZJU01/2013(H7N9). The different degrees of injury at 2, 4, and 6 days after virus inoculation are shown (original magnification, 200×). C: The H7 gene content in lung tissue. D: The TCID_50_ of the lung leachate. E: The lungs of mice challenged with A/Guangdong/GZ8H002/2017(H7N9). The different degrees of injury at 2, 4, and 6 days after virus inoculation (200×). HE: Hematoxylin eosin staining; IHC: Immunohistochemical staining; dpi: days post infection.

The histopathology of organ tissues was evaluated using HE staining. The extent and characteristics of the lesions varied among the groups. The mice infected with A/Zhejiang/DTID-ZJU01/2013(H7N9) had multifocal interstitial inflammatory hyperaemia and exudative pathological changes in the lung, with larger lesions in the lung tissue and fusion of multiple patchy lesions. These histopathological changes were severe at 2 dpi. In the following days, the pathological changes of the lung tissue persisted, but gradually improved. At 6 dpi, the pathological change became moderate. This was consistent with the improvement in the mice’s general health (Figure 4A,B). The pulmonary pathological changes of mice infected with A/Guangdong/GZ8H002/2017(H7N9) appeared relatively late, but progressed continuously. At 2 dpi, the mice showed mild pulmonary injury. However, at 4 dpi, the pathological changes in the lungs were severe. The pulmonary pathological changes continued to worsen until death (Figure 4E). Immunohistochemical staining of lungs showed various degrees of injury at 2, 4, and 6 dpi (Figure 4A, B, E). Viral antigens could be detected in the lung, especially in the bronchiolar epithelium.

High levels of the H7 virus gene were detected persistently in the lungs of the H7N9-infected mice (Figure 4C). At 2 dpi the expression of the H7 gene was higher in the A/Zhejiang/DTID-ZJU01/2013(H7N9)-infected mice than in the A/Guangdong/GZ8H002/2017(H7N9)-infected mice. However, as infection continued, the expression of H7 gene in the A/Guangdong/GZ8H002/2017(H7N9)-infected mice increased rapidly. At 4 and 6 dpi, the expression of the H7 gene in the A/Guangdong/GZ8H002/2017(H7N9) infection group was significantly higher than that in the A/Zhejiang/DTID-ZJU01/2013(H7N9) infection group. The TCID50 was persistently detected in lung (Figure 4C). At 4 and 6 dpi, the TCID50 was higher in the A/Guangdong/GZ8H002/2017(H7N9) infection group than in the A/Zhejiang /DTID-ZJU01/2013(H7N9) infection group. H7N9 virus was isolated persistently from the lung tissue collected at 2, 4, and 6 dpi in both infection groups (Table 2).

### Histopathology of brain tissue and the amount of virus in brain tissue

**Figure 5.**
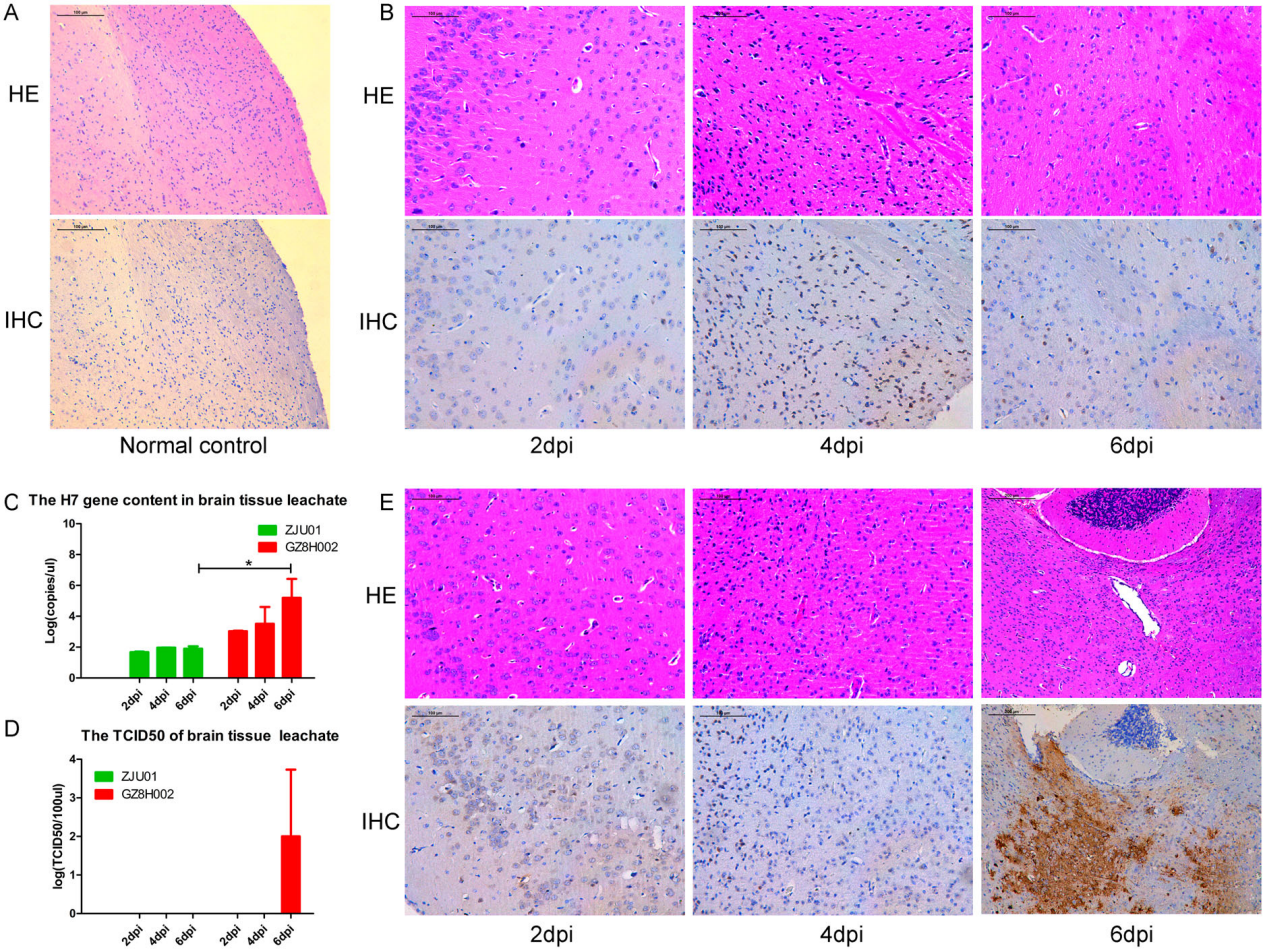
Histopathology of brain tissue and the brain viral titre. A: HE staining and immunohistochemical staining of brain samples from normal mice. B: The brain of mice challenged with A/Zhejiang/DTID-ZJU01/2013(H7N9). The different degrees of injury at 2, 4, and 6 days after virus inoculation (original magnification, 200×). C: The H7 gene content in brain tissue. D: The TCID50 of the brain leachate. E: The brain of mice challenged with A/Guangdong/GZ8H002/2017(H7N9). The different degrees of injury at 2, 4, and 6 days after virus inoculation (200×). HE: Hematoxylin eosin staining; IHC: Immunohistochemical staining; dpi: days post infection.

In both infection groups, HE staining of brains presented inflammatory cell infiltration (Figure 5B, E). In the A/Zhejiang/DTID-ZJU01/2013(H7N9)-infected mice, moderate brain pathology was observed (Figure 5B). By contrast, the brains of the mice infected with A/Guangdong/GZ8H002/2017(H7N9) presented increasingly severe pathological changes with extended infection time. In IHC staining of brains, large amounts of H7N9 viral antigens were detected in the brain at 6 d after infection (Figure 5E). The results suggested that the virus had infected the brain tissue.

We also detected the expression of the H7 gene in brain leachate (Figure 5C). The H7 gene was detected persistently in both infection groups. The H7 gene level in A/Zhejiang /DTID-ZJU01/2013(H7N9)-infected mice were stable at 2, 4, and 6 dpi. However, in the A/Guangdong /GZ8H002/2017(H7N9)-infected mice, the amount of H7 gene detected at 6 dpi increased dramatically. The level of H7 gene was significantly higher than that in the A/Zhejiang/DTID-ZJU01/2013(H7N9)-infected mice (Figure 5C).

The TCID50 of brain leachate was only detected at 6 dpi in the A/Guangdong/GZ8H002/2017(H7N9)-infected mice (Figure 5D). In the A/Guangdong/GZ8H002/2017(H7N9)-infected mice, live virus was isolated from the brain leachate at 6 dpi. However, the H7N9 virus could not be isolated from the brain leachate of the A/Zhejiang/DTID-ZJU01/2013(H7N9)-infected mice (Table 2).

### H7n9 virus detection in exosomes

**Figure 6.**
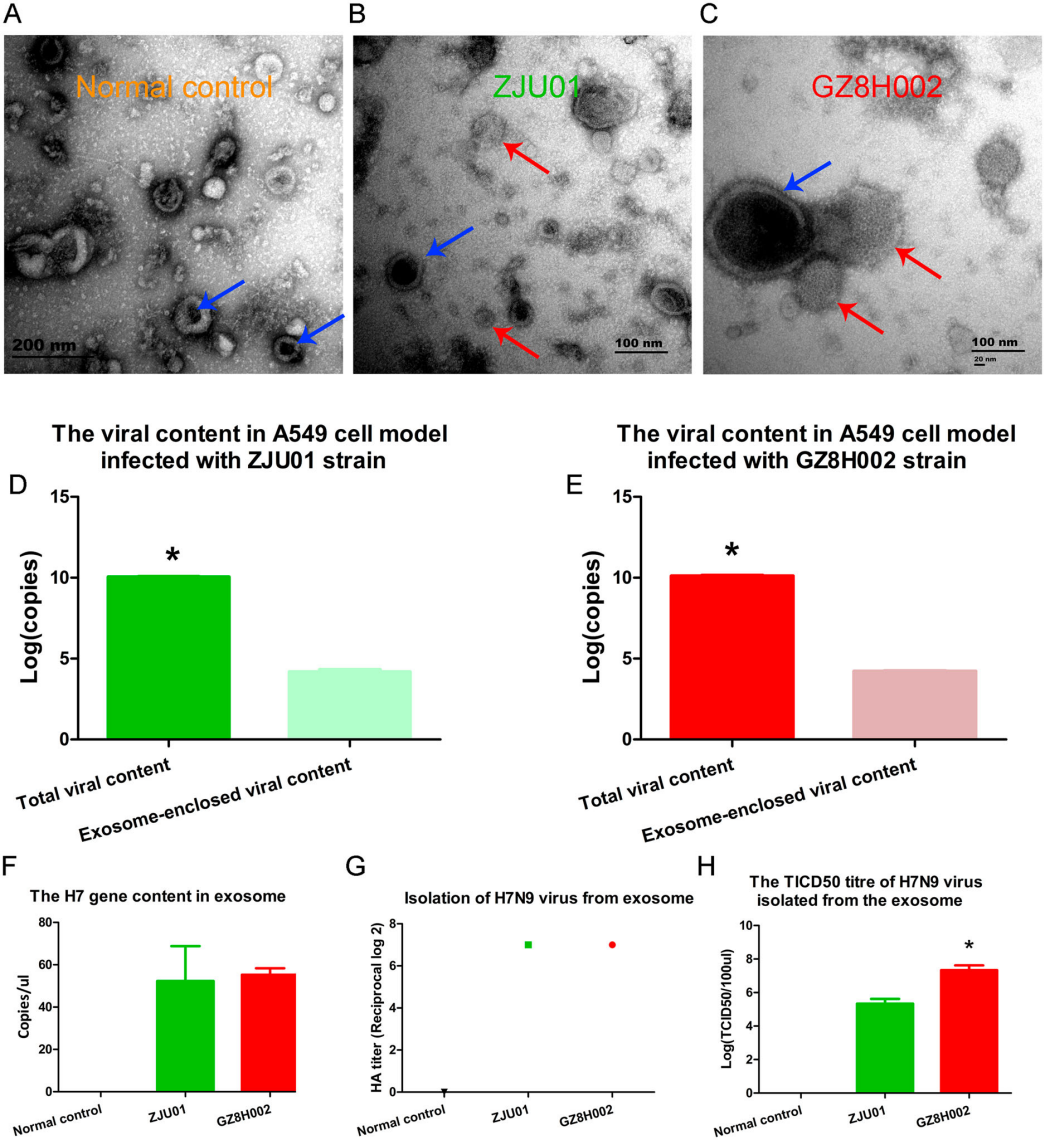
Isolation of exosomes and identification of viral genes. A: The culture supernatant from A549 cell model without virus infection was observed using transmission electronic microscope. The exosomes were showed by blue arrow. B: The culture supernatant from A549 cell model with ZJU01 strain infection was observed using transmission electronic microscope. The exosome was showed by blue arrow. The viral particles were viewed and showed by red arrow. C: The culture supernatant from A549 cell model with GZ8H002 strain infection was observed using transmission electronic microscope. The exosome was showed by blue arrow. The viral particles were viewed and showed by red arrow. D: The viral content in A549 cell model infected with ZJU01 strain. E: The viral content in A549 cell model infected with GZ8H002 strain. F: The H7 gene content in exosomes. G: The isolation of H7N9 virus from exosomes. H: The TCID50 titre of the H7N9 virus isolated from the exosomes.

The exosomes were enriched using ultra-centrifugation. The morphology of the exosome was viewed using TEM. The typical shape of exosomes was observed in the different groups. The shape and size of the exosome varied from 50 to 200 nm. The culture supernatant from A549 cell model without virus infection was observed using transmission electronic microscope. The exosomes were showed by blue arrow (Figure 6A). The culture supernatant from A549 cell model with ZJU01 strain infection was observed using transmission electronic microscope. The exosome was showed by blue arrow. The viral particles were viewed and showed by red arrow (Figure 6B). The culture supernatant from A549 cell model with GZ8H002 strain infection was observed using transmission electronic microscope. The exosome was showed by blue arrow. The viral particles were viewed and showed by red arrow (Figure 6C). We found the exosome was closely related to virus. The virus seemed to be released from the exosome. This may be the direct evidence that exosomes contain viral particles.

After purification, the H7N9 virus genes in the exosomes were detected using quantitative PCR. The exosome-enclosed viral particle is a very small part compared to the total viral particle. The H7 gene content of total viral particle and exosome-enclosed viral particle were presented in Figure 6D,E. The H7N9 amplicons were detected in samples of exosomes from both A/Zhejiang/DTID-ZJU01/2013(H7N9) or A/Guangdong/GZ8H002/2017(H7N9) infected cells (Figure 6F). We also isolated the live virus from the exosomes (Figure 6G). The TCID50 titre of GZ8H002 was higher than ZJU01(Figure 6H).

### Working model of viraemia and extrapulmonary infection

**Figure 7.**
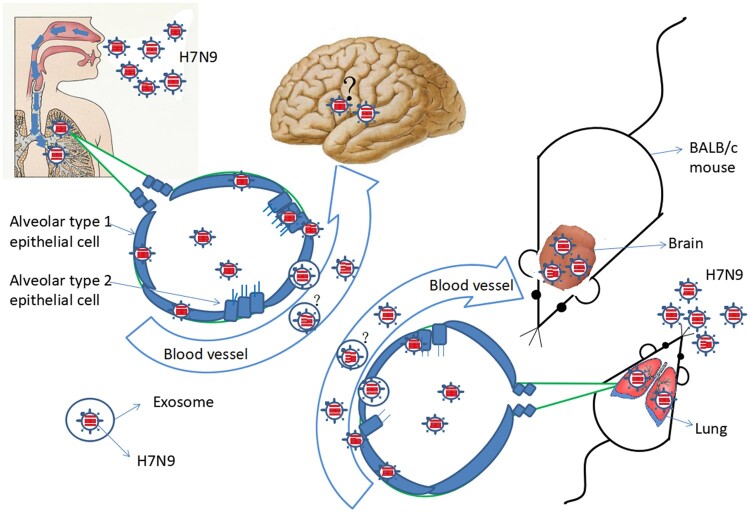
Working model of viraemia and extrapulmonary infection by HPAI H7N9.

HPAI H7N9 virus infection starts in the respiratory tract. After infection, HPAI H7N9 virus replicates in the lung. Meanwhile, the patient suffered from persistent viraemia and a cytokine storm was observed his plasma. Live H7N9 virus was detected the patient’s blood. These phenomena were confirmed using animal experiments. How is the virus transmitted from the lung to the blood? One possible explanation is that inflammation leads to the disruption of the lung-blood barrier. Another possibility is that the virus is released into the blood through via exosomes. When the H7N9 virus exists in the blood, the virus might cause extrapulmonary infection. We speculated that the virus could infect the brain, after observing this phenomenon in the mouse model (Figure 7). However, we could not confirm this because of the lack of brain tissue from the deceased patient.

## Discussion

In the present study, HPAI H7N9 infection of a patient proved to be deadly. This patient presented with viraemia and died 3 days after admission. Whether there is live virus in the blood and whether it could lead to extrapulmonary infection have been two important questions. Previous studies detected viral RNA from LPAI H7N9, H5N1, and H1N1 infections [5,6,8]. H5N1 can be transmitted in ferrets by transfusion [30]. However, there has been no report of the isolation of the virus from the blood of patients with H7N9 infections. We successfully isolated the live virus from the patient’s plasma. We confirmed that the A/Guangdong/GZ8H002/2017(H7N9) virus persisted in the blood with mouse experiments.

In the previous studies of H5N1 and pandemic 2009 H1N1 virus infection, viraemia was commonly existed in the fatal cases, but was absent in nonfatal cases [5,6]. In previous study of H7N9, the virus RNA could only be detected in the lethal cases [8]. The amount of RNA detected in H7N9 patients is very small, and there is no previous report about patient with H7N9 infection to be confirmed to have viraemia. While in highly pathogenic H7N9 infection the viraemia was confirmed, which makes a great difference. At present, the mortality rate of H7N9 is about 40%, while as reported the mortality rate of highly pathogenic H7N9 is as high as 50% [1,2]. We speculated that there is a relationship between viraemia and high mortality. But more experiments still need to be carried out to confirm this suggestion.

In our previous study, we observed that the levels of 34 of the 48 cytokines and chemokines tested were significantly elevated in plasma samples from patients infected with LPAI H7N9. We further found that the levels of MIF, SCF, MCP-1, HGF, and SCGF-β were highly positively correlated to disease severity. In addition, the profile of mediators MIF, SCF, MCP-1, HGF, SCGF-β, IP-10, IL-18, and IFN-γ is an independent outcome predictor [31]. Cytokinemia was also observed in patients with H5N1 infection. The levels of IL-10, IL-6, and IFN-γ were elevated in H5N1-infected individuals [5]. In the present study, 32 cytokines and chemokines were significantly elevated in the patient. Among them, the levels of ten cytokines and chemokines, including MIG, IL-1ra, IL-6, IP-10, IL-10, IFN-γ, MCP-3, IL-18, HGF, and MCP-1, were dramatically higher (> 30-fold) in this patient compared with those in the healthy controls. The acute cytokinemia might lead to the patient’s death. The similar phenomenon was also observed in the mouse experiment. The mice infected with A/Guangdong/GZ8H002/2017(H7N9) suffered a persistent cytokinemia, which might be the cause of death.

Influenza virus infection of the respiratory tract is associated with a range of neurological complications. The incidence of severe pandemic H1N1 influenza-associated neurological complications was estimated to be 1.2 per 1,00,000 persons among children and Asian Pacific patients [32,33]. The H1N1 influenza virus has not been detected in patients’ cerebrospinal fluid or brain tissue [34]. However, in a mouse experiment, H1N1 was found to infect many brain regions and induce neuropathological changes in neonatal mouse brains [35]. Thus, Influenza virus has the possible ability to infect the brain [36]. In an *in vitro* study, avian H7N9 virus could effectively infect human brain cells. Meanwhile, the virus could be transcribed, replicate its viral genome, and generate infectious progeny virus [37]. In the present study, we detected relatively high levels of virus RNA in brain of mice infected with A/Guangdong/GZ8H002/2017(H7N9) and A/Zhejiang/DTID-ZJU01/2013(H7N9) virus. However, in the TCID50 test of the brain leachate, no H7N9 virus was found in the samples of mice infected with the A/Zhejiang/DTID-ZJU01/2013(H7N9) virus. By contrast, the TCID50 test was positive for A/Guangdong/GZ8H002/2017(H7N9) in the brain leachate at 6 dpi. The brain pathological changes supported this finding. We identified high levels of virus replication in the brain. Furthermore, we isolated live virus from the brain leachate. The replication of virus in the brain could cause profound central nervous system injury. Observation of neurological changes caused by H7N9 virus infection deserves further attention when managing these patients.

However, we must say that we haven’t fully revealed the potential mechanism behind this feature of the 2017 H7N9 strain. But we observed the GZ8H002 strain might have acquired the possible key molecular substitutions associated with increased virulence and transmissibility in mammals. As we know, the presence of KRTA insertion in the cleavage sites enhanced the extrapulmonary infection ability and virulence of HPAI virus [38,39]. This effect has been also confirmed in H5N1 and H7N7 [40,41]. Influenza related encephalitis has also been found in H5N1 infection [42,43]. The mutation of K526R is related to the increase of virus replication ability and the mutation of M535L may increase the virus polymerase activity [44–46]. We found GZ8H002 strain has both K526R and M535L mutations. On the basis of the insertion of KRTA at HA cleavage site of highly pathogenic H7N9, cumulative effect may occur, which together leads to the enhancement of pathogenicity and neurotropism of highly pathogenic H7N9. But, more experiments are needed to confirm whether these mutations are essential for high virulence of highly pathogenic H7N9 virus.

Exosomes are cellular secreted vesicles that are free to enter cells in a receptor-independent manner. Exosomes have been found to be closely associated with viral infection [10], and can carry large amounts of viral nucleic acid or protein components. Studies have demonstrated that exosomes can carry HIV, hepatitis C virus, HAV, and Zika virus, causing the virus to spread [12–14,16]. In the present study, we used the A549 cell model and found that A549 cells secreted exosomes under viral pressure. Gene fragments of HPAI H7N9 virus were detected in these exosomes. In addition, after inoculating chicken embryos with these exosomes, we isolated the H7N9 virus, further supporting the hypothesis that exosomes carry the entire genome of the H7N9 virus or live virus. These findings suggested that H7N9 extrapulmonary infection might develop via the exosome pathway. However, confirmatory trials are required to further clarify whether exosomes are indeed a route to extrapulmonary infection.

In summary, the viraemic patient was confirmed to be infected with HPAI H7N9. Cytokine storms and potential brain infections might be the cause of patient death. Exosomes might be the pathway that leads to infection in extrapulmonary tissues.

## Abbreviations

**ABBREVIATION**: RT–PCR: Reverse transcription polymerase chain reaction; MOI: The multiplicity of infection; HPAI: Highly pathogenic avian influenza; LPAI: Low-pathogenic avian influenza; HIV: Human immunodeficiency virus; HAV: Hepatitis A virus; HCV: Hepatitis C virus; SPF: Specific-pathogen-free; MDCK: Madin-Darby canine kidney cell line; SNPs: Single nucleotide polymorphisms; INDEL: Insertion/deletion; TCID50: Fifty percent tissue culture infective dose; PBS: Phosphate-buffered saline; TEM: Transmission electron microscopy; Dpi: Days post infection; TPCK: L-l-tosylamide-2-phenylethyl chloromethyl ketone; PDGF-BB: Platelet Derived Growth Factor-BB; G-CSF: Granulocyte colony stimulating factor; SCGF-β: Stem Cell Growth Factor-β; SCF: Stem Cell Factor; MIF: Macrophage Migration Inhibitor Factor; MIP: Macrophage Inflammatory protein; IL-1ra: Interleukin-1 receptor antagonist; IL-2Rα: Interleukin-2 receptor alpha; IP-10: Interferon-inducible protein 10; IFN-γ: Interferon γ; MCP: Monocyte Chemotactic Protein; HGF: Hepatocyte Growth Factor; MIG: Monokine induced by interferon-gamma; IL-12(p40): Interleukin-12 (p40); CTACK: Cutaneous T-Cell Attracting Chemokine; GM-CSF: Granulocyte-macrophage colony stimulating factor; RANTES: Regulated upon activation, normal T-cell expressed and secreted; TNF-α: Tumor Necrosis Factor-α; FGF basic: Basic fibroblast growth factor; SDF-1α: Stromal cell derived factor 1α; TRAIL: TNF-related apoptosis-inducing ligand; KC: Keratinocyte derived chemokine; RNA: Ribonucleic Acid; FBS: Fetal bovine serum; BSA: Bovine serum albumin; ZJU01: A/Zhejiang/DTID-ZJU01/2013(H7N9); GZ8H002: A/Guangdong/GZ8H002/2017(H7N9); HA: Hemagglutinin; NA: Neuraminidase; PA: Polymerase acidic protein; PB1: Polymerase basic protein 1; PB2: Polymerase basic protein 2; NP: Nucleocapsid protein; MP: Matrix protein; NS: Non-structural protein; cDNA: Complementary deoxyribonucleic acid.
